# ﻿New leafhopper species and new records of Typhlocybini (Hemiptera, Cicadellidae, Typhlocybinae) from China

**DOI:** 10.3897/zookeys.1082.73611

**Published:** 2022-01-20

**Authors:** Xian Zhou, Yalin Zhang, Min Huang

**Affiliations:** 1 Key Laboratory of Plant Protection Resources and Pest Management of Ministry of Education, Entomological Museum, College of Plant Protection, Northwest A&F University, Yangling, Shaanxi Province, 712100, China Northwest A&F University Yangling China

**Keywords:** Auchenorrhyncha, distribution, morphology, taxonomy, Typhlocybini

## Abstract

Five genera from China of the leafhopper tribe Typhlocybini are treated. *Linnavuoriana* Dlabola, 1958 and *Shamala* Dworakowska, 1980 and seven known species, *Edwardsianacorylicola* Vilbaste, 1968, *E.praedestina* Dlabola, 1967, *E.singularis* Anufriev, 1975, *Hiratettixdistanti* Dworakowska, 1982, *H.malaisei* Dworakowska, 1982, *L.antiqua* Dworakowska, 1982, and *L.malicola* Zachvatkin, 1949 are newly recorded from China. Two new species, *Shamalaannulata* and *Paracybabiprocessa***spp. nov.**, are described and illustrated. Keys to Chinese species of each genus are also provided.

## ﻿Introduction

Typhlocybini (Hemiptera, Cicadellidae, Typhlocybinae) is a moderately large leafhopper tribe with over 924 species in 93 genera worldwide, of which 253 species in 44 genera have been recorded from China (including Zyginellini) (Dmitriev 2003; Yan 2019). Recent studies on Typhlocybinae from China have revealed many new taxa and new records, especially in the tribe Typhlocybini. Here, we treat nine species belonging to five genera of this tribe, including two new species which are described and illustrated and seven new records. Updated keys to Chinese species of each genus are also provided.

## ﻿Materials and methods

Figures of the specimens were made using a Leica M205 light microscope with a Leica DFC425 camera. Images were produced using the Leica Application Suite V3.7 and edited using Adobe Photoshop CS6.0 (Adobe Systems). Abdomens were removed from examined specimens and macerated in cold 10% NaOH solution overnight, subsequently rinsed for 30 s with pure water, and stored in glycerin. An Olympus SZX10 microscope was used for dissecting specimens and an Olympus PM-10AD was used for drawing the dissected male genitalia.

Morphological terminology in this work follows Zhang (1990), but wing venation follows Dworakowska (1993).

Type specimens of the new species are deposited in the collections of the Entomological Museum, Northwest A&F University, Yangling, China (**NWAFU**).

## ﻿Taxonomy

### ﻿ Edwardsiana

Taxon classificationAnimaliaHemipteraCicadellidae

Zachvatkin

8B03528D-C035-58FA-A0B8-62D3365FFCD6

Edwardsiana Zachvatkin, 1929: 439.

#### Type species.

*Cicadarosae* Linnaeus, 1758, by original designation.

#### Remarks.

The genus *Edwardsiana* includes 80 known species worldwide (Dmitriev 2003), with two species having been reported from China. Here we record three more species from China and provide a key to all Chinese species.

#### Diagnosis.

**Figures 1–20.**
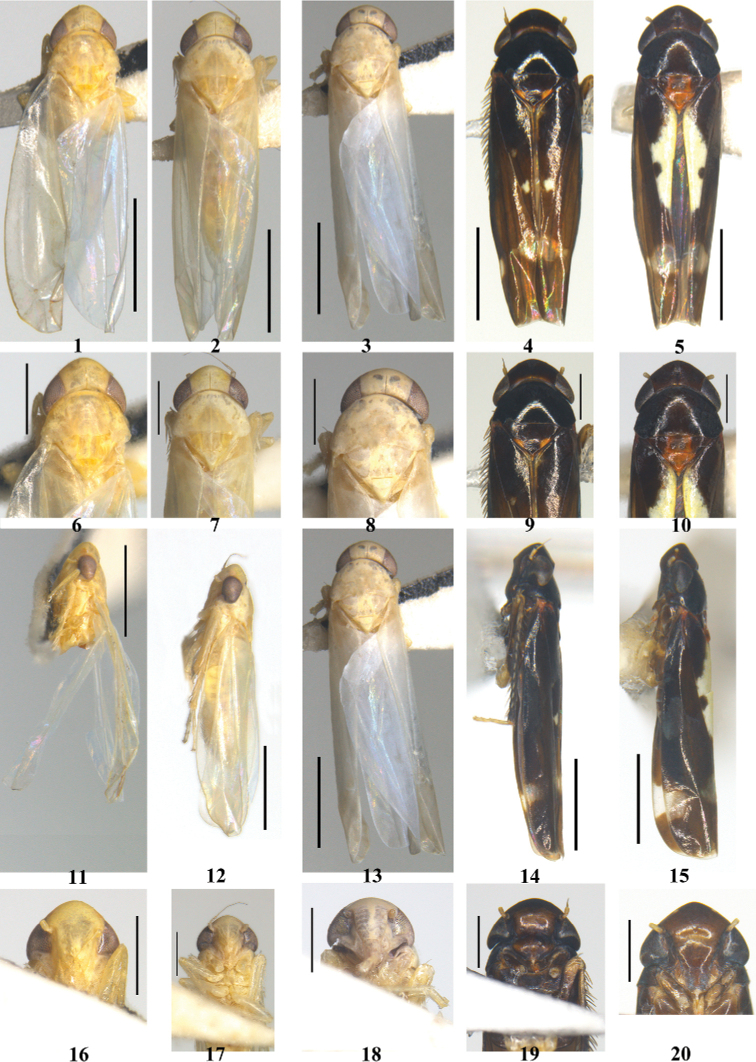
Typhlocybini of China **1–5** dorsal view **6–10** head and thorax, dorsal view **11–15** lateral view **16–20** face **1, 6, 11, 16***Edwardsianacorylicola***2, 7, 12, 17***E.praedestina***3, 8, 13, 18***E.singularis***4, 9, 14, 19***Hiratettixdistanti***5, 10, 15, 20***H.malaisei*. Scale bars: 1.0 mm (**1–5, 11–15**); 0.5 mm (**6–10, 16–20**).

Body cream with variable patches (Figs 1–3). Crown bluntly produced, medial length shorter than distance between eyes; coronal suture distinct. Pronotum slightly wider than head (Figs 6–8). Forewing with apical area short, 1/4–1/3 of total length; RP and MP’ petiolate or not. Hind wing with R and M confluent distally.

Male sternal abdominal apodemes well developed, often extending to middle of 6^th^ sternite.

**Male genitalia.** Pygofer side often with rounded extension at basal angle; ventral part always with depressed areas, dense stout setae on ventral-basal part and row of short rigid setae caudally. Subgenital plate elongate with subapical part twisted outwards; long macroseta basally and row of short rigid setae from middle to subapex. Paramere with distal part long and curved. Connective with central ridge developed. Aedeagus with preatrium and dorsal apodeme developed; aedeagal shaft with paired apical processes; gonopore apical.

#### Distribution.

Palaearctic and Nearctic regions.

### ﻿Key to species (males) of the genus *Edwardsiana* from China

**Table:** 

1	Aedeagal shaft with two pairs of unbranched apical processes	***E.rosae* (Linnaeus)**
–	Aedeagal shaft with apical processes branched	**2**
2	Aedeagal shaft with both dorsal and ventral processes branched (Figs 41, 42)	***E.singularis* Anufriev**
–	Aedeagal shaft just with dorsal processes branched	**3**
3	Ventral processes of aedeagal shaft short and directed ventrad (Figs 37, 38)	***E.corylicola* Vilbaste**
–	Ventral processes of aedeagal shaft long and directed dorsad	**4**
4	Aedeagal shaft with dorsal and ventral processes branched near base (Figs 39, 40)	***E.praedestina* Dlabola**
–	Aedeagal shaft with dorsal and ventral processes branched at middle or near apex	***E.ishidai* (Matsumura)**

### ﻿ Edwardsiana corylicola

Taxon classificationAnimaliaHemipteraCicadellidae

Vilbaste rec. nov.

9874680D-1881-541B-A20E-1035AC5EC5A1

Figs 1 , 6 , 11 , 16 , 37 , 38

Edwardsiana corylicola Vilbaste, 1968: 98, Dworakowska 1982: 121, figs 205, 206.

#### Specimens examined.

2♂♂, 2♀♀, China, Heilongjiang Province, Mishan, 2.ix.2001, coll. Qiang Sun.

#### Distribution.

China (Heilongjiang), Russia, Korea, North Korea.

### ﻿ Edwardsiana ishidai

Taxon classificationAnimaliaHemipteraCicadellidae

(Matsumura)

3EFEC1F8-2F27-57E4-ABED-D4F43AA2EE15

Typhlocyba ishidai Matsumura, 1932: 98, pl. II, fig. 3a, b.
Typhlocyba lanternae Wagner, 1937: 154.
Edwardsiana ussurica Vilbaste, 1968: 97.
Edwardsiana ishidai (Matsumura): Dworakowska 1982: 112, figs 156–178; Huang and Zhang 2002: 290.

#### Distribution.

China (Jilin), Japan, Mongolia, Russia.

### ﻿ Edwardsiana praedestina

Taxon classificationAnimaliaHemipteraCicadellidae

Dlabola rec. nov.

45374B59-3E2E-50A1-876C-F690A090320F

Figs 2 , 7 , 12 , 17 , 39 , 40

Edwardsiana praedestina Dlabola, 1967: 217, figs 12–14.

#### Specimens examined.

1♂, 19♀♀, China, Shandong Province, Kunyu Mountain, 12.vii.2001, coll. Daozheng Qin and Zhenjiang Liu.

#### Distribution.

China (Shandong), Mongolia.

### ﻿ Edwardsiana rosae

Taxon classificationAnimaliaHemipteraCicadellidae

(Linnaeus)

060FDEDC-F192-5FEE-95B2-40A0CB80DBD7

Cicada rosae Linnaeus, 1758: 439.
Edwardsiana subrosea Vilbaste, 1980: 41.
Edwardsiana rosae (Linnaeus): Dworakowska 1982: 107, figs 106–117.

#### Distribution.

China (Gansu, Xinjiang), Cyprus, Turkey, Iran, Russia, Kazakhstan, Kirghizstan, Uzbekistan, Tajikistan.

### ﻿ Edwardsiana singularis

Taxon classificationAnimaliaHemipteraCicadellidae

Anufriev rec. nov.

B5797010-2EBE-59AB-AC90-F0216A55E77D

Figs 3 , 8 , 13 , 18 , 41 , 42

Edwardsiana singularis Anufriev, 1975: 531; Dworakowska 1982: 117, figs 216, 217.

#### Specimens examined.

1♂, China, Heilongjiang Province, Mishan, 2.ix.2001, coll. Qiang Sun.

#### Distribution.

China (Heilongjiang), Russia, Kazakhstan.

### ﻿ Hiratettix

Taxon classificationAnimaliaHemipteraCicadellidae

Matsumura

B402D3C0-F8CA-5804-A0C7-033F2FB4BE6A

Hiratettix Matsumura, 1931: 59 (in key); Matsumura 1932: 102 (full description); Dworakowska 1982: 152.

#### Type species.

*Hiratettixarisanellus* Matsumura, 1932.

#### Remarks.

After Matsumura (1931) described the genus *Hiratettix* (in a key to genera), Dworakowska (1982) added three new species from China (Taiwan), Myanmar, and India, and Sohi et al. (1990) published a new species from Nepal. Here we report two species new to China and provide a key to the Chinese species.

#### Diagnosis.

Body flat and overall black (Figs 4, 5). Face wide and short (Figs 19, 20). Pronotum with minute transverse sculpture (Figs 9, 10). Forewing with 3^rd^ apical cell triangular. Hind wing truncated terminally with two cross veins; apical cells short.

**Male genitalia.** Genital capsule high and short. Pygofer with several short rigid setae terminally on inner surface and long macrosetae ventrobasally. Subgenital plate broad basally, slightly narrowed distally, with several long macrosetae near middle part, a row of short rigid setae laterally and progressively shorter subbasally to apex. Connective wide with central lobe underdeveloped. Paramere thick with central part longer than basal and distal parts together; caudal part with sensorial pits on inner margin. Aedeagal shaft with paired apical and lateral processes; gonopore apical.

#### Distribution.

Oriental Region.

### ﻿Key to species (males) of the genus *Hiratettix* from China

**Table:** 

1	Aedeagal shaft with two long basal processes and two pairs of short apical processes	**2**
–	Aedeagal shaft with two medium sized lateral processes and one pair of short apical processes	**3**
2	Aedeagal shaft with basal processes S-bent and two pairs of apical processes basally closed	***H.arisanellus* Matsumura**
–	Aedeagal shaft with basal processes arched and two pairs of apical processes detached from each other basally	***H.matsumurai* Dworakowska**
3	Aedeagal shaft with median processes curved outwards, apical processes directed to each other distally (Figs 43, 44)	***H.distanti* Dworakowska**
–	Aedeagal shaft with median processes straight, apical processes widely separated distally (Figs 45, 46)	***H.malaisei* Dworakowska**

### ﻿ Hiratettix arisanellus

Taxon classificationAnimaliaHemipteraCicadellidae

Matsumura

832E14D7-7409-5974-B00B-D64AEA9E5B64

Hiratettix arisanellus Matsumura, 1932: 102; Dworakowska 1982:153, figs 635–649.

#### Distribution.

China (Taiwan).

### ﻿ Hiratettix distanti

Taxon classificationAnimaliaHemipteraCicadellidae

Dworakowska rec. nov.

BF4DF362-A8CA-5135-BD73-1149E21EA030

Figs 4 , 9 , 14 , 19 , 43 , 44

Hiratettix distanti Dworakowska 1982: 154, figs 661–666.

#### Specimens examined.

1♂, China, Yunnan Province, Mengla, Nangong Mountain, 1850 m, 13.xii.1999, coll. Dworakowska.

#### Distribution.

China (Yunnan), India.

### ﻿ Hiratettix malaisei

Taxon classificationAnimaliaHemipteraCicadellidae

Dworakowska rec. nov.

DE486C12-9C43-5042-8BEF-5307A87B106F

Figs 5 , 10 , 15 , 20 , 45 , 46

Hiratettix malaisei Dworakowska 1982: 153, figs 667–676.

#### Specimens examined.

1♂, China, Sichuan Province, Emei Mountain, 950 m, 30.x.1999, coll. Dworakowska. 1♂, 2♀♀, China, Guizhou Province, Huaxi, Huaxi Garden, 1100 m, 25.vii.2001, coll. Qiang Sun, at light. 1♂, 14♀♀, China, Yunnan Province, Shilin, 9.vii.2021, coll. Xian Zhou.

#### Distribution.

China (Sichuan, Guizhou, Yunnan), Myanmar.

### ﻿ Hiratettix matsumurai

Taxon classificationAnimaliaHemipteraCicadellidae

Dworakowska

44FAA73C-27FF-5A04-8B57-3536C8BA45CB

Hiratettix matsumurai Dworakowska 1982: 153, figs 650–660.

#### Distribution.

China (Taiwan).

### ﻿ Linnavuoriana

Taxon classificationAnimaliaHemipteraCicadellidae

Dlabola rec. nov.

B9B66579-2B48-5D81-A3AF-CC83293B80E9

Linnavuoriana Dlabola 1958: 54; Dworakowska 1971: 647; Dworakowska 1982: 121.

#### Type species.

*Cicadadecempunctata* Fallen, 1806.

#### Remarks.

Up to now, there are eight known species in the genus *Linnavuoriana*. In Xinjiang and Yunnan provinces, we collected two known species that are the first records of the genus from China. A key to the Chinese species is given.

#### Diagnosis.

**Figures 21–36.**
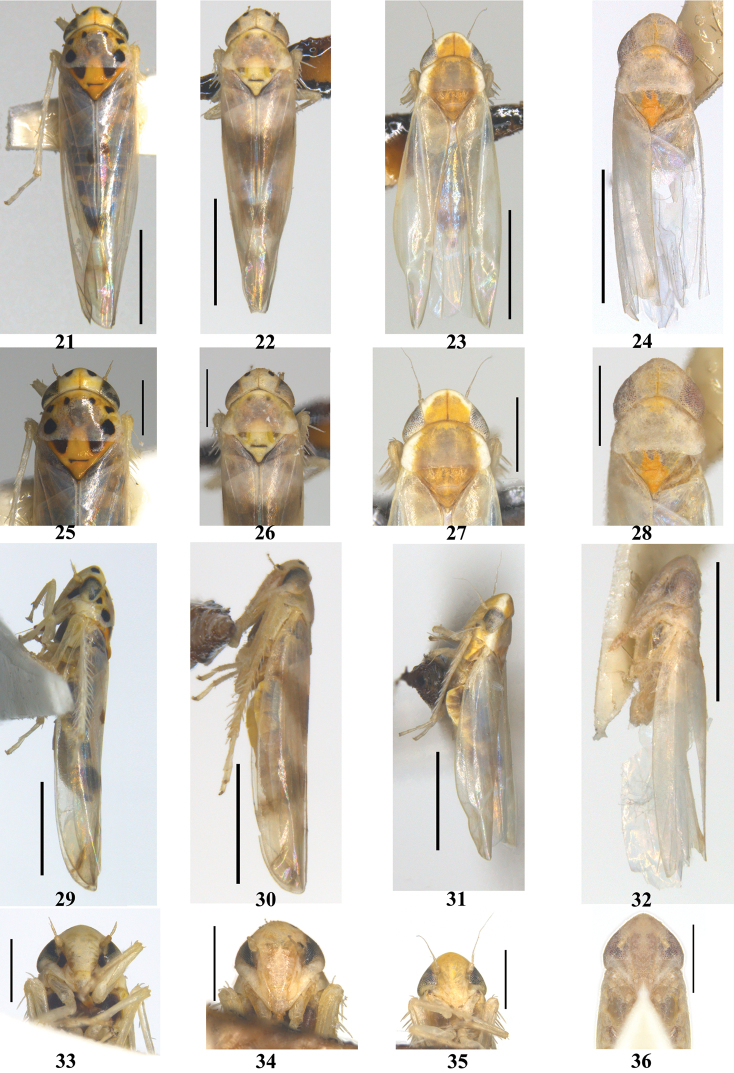
Typhlocybini of China **21–24** dorsal view **25–28** head and thorax, dorsal view **29–32** lateral view **33–36** face **21, 25, 29, 33***Linnavuorianaantiqua***22, 26, 30, 34***L.malicola***23, 27, 31, 35***Paracybabiprocessa* sp. nov. **24, 28, 32, 36***Shamalaannulata* sp. nov. Scale bars: 1.0 mm (**21–24, 29–32**); 0.5 mm (**25–28, 33–36**).

Body cream, light yellow (Figs 21, 22). Crown and pronotum with symmetrical black round patches, and basal triangle black (Figs 25, 26). Forewing semitransparent with light smoky brown or brown patches (Figs 21, 22, 29, 30).

Head bluntly produced, little narrower than width of pronotum, and 1/3 times middle length of pronotum (Figs 25, 26). Forewing slender and obtuse apically with both sides parallel; RP+MP’ petiolate at base; 1^st^ apical cell usually smallest; 2^nd^ apical cell biggest. Hind wing with two cross veins far away from each other.

Abdominal apodemes well developed, often extending to 5^th^ abdominal sternite.

**Male genitalia.** Hind margin of pygofer with inner ridge, several rigid microsetae near posterior-dorsal margin and numerous minute tubercles posteroventrally. Subgenital plate parallel-sided and lack macrosetae at base, narrowing from apical 1/3–1/2 of outer margin, rounded terminally, usually with row of rigid microsetae and some fine microsetae scattered on apex. Paramere rarely curved with subapical tooth; distally with row of microsetae on outer margin and row of sensorial pits on inner margin. Connective small with central ridge underdeveloped. Aedeagal shaft with a pair of triangular protrusions on dorsal surface laterally; gonopore apical.

#### Distribution.

Oriental and Palaearctic regions.

### ﻿Key to species of *Linnavuoriana* from China (males)

**Table:** 

1	Crown and pronotum with large dark patches; aedeagal shaft with apex hook-like (Figs 47, 48)	***L.antiqua* Dworakowska**
–	Crown and pronotum with few light patches; aedeagal shaft with apex not hook-like (Figs 49–54)	***L.malicola* (Zachvatkin)**

### ﻿ Linnavuoriana antiqua

Taxon classificationAnimaliaHemipteraCicadellidae

Dworakowska rec. nov.

B6FF126B-2595-561D-8327-033F7BAFC619

Figs 21 , 25 , 29 , 33 , 47 , 48

Linnavuoriana antiqua Dworakowska 1982: 123, figs 326–341.

#### Specimens examined.

1♂, 1♀, China, Yunnan Province, Lijiang, Xinzhu Botanical Garden, 16.xi.1999, coll. Dworakowska. 1♂, Yunnan Province, Tengchong, 1650 m, 26.iv.1981, coll. Fasheng Li.

#### Distribution.

China (Yunnan), India.

### ﻿ Linnavuoriana malicola

Taxon classificationAnimaliaHemipteraCicadellidae

(Zachvatkin) rec. nov.

FFC4FC2F-D125-5541-92C7-70BADC98B7C8

Figs 22 , 26 , 30 , 34 , 49–54

Typhlocyba malicola Zachvatkin, 1949: 220.
Linnavuoriana taschkentica Dlabola, 1961: 302, figs 152–156. Synonymized by Mitjaev 1967: 710.
Linnavuoriana apunctata Mitjaev, 1963: 49, nec Dlabola.
Typhlocyba roseipennis Kusnezov, 1932: 232, nec Oshanin (Zachvatkin 1949;Mitjaev 1967).

Linnavuoriana populicola Dubovsky, 1966: 132; Synonymized by Dworakowska 1982: 123, figs 313–325.

#### Specimens examined.

4♂♂, 3♀♀, China, Xinjiang Province, Xinjiang Agricultural Vocational and Technical College, 16.ix.1986, coll. Yalin Zhang. 13♂♂, 4♀♀, Xinjiang Agricultural University, 16.ix.1986, coll. Yalin Zhang.

#### Distribution.

China (Xinjiang), Kazakhstan, Kirghizstan, Uzbekistan, Tajikistan, Afghanistan.

### ﻿ Paracyba

Taxon classificationAnimaliaHemipteraCicadellidae

Vilbaste

97108883-5D97-562E-B054-19D67E336215

Paracyba Vilbaste, 1968: 96; Dworakowska 1982: 148, figs 602–621.

#### Type species.

*Zyginaakashiensis* Takahashi, 1928.

#### Remarks.

Until now, the genus *Paracyba* included three known species, including two species from China. Here we add a new species from China and give a key to all species of the genus.

#### Diagnosis.

Body slim. Head bluntly produced, middle length equal to or shorter than width between eyes, coronal suture long and distinct. Forewing laterally with apex rounded; RP and MP’ petiolate at base; 1^st^ apical cell nearly equal in size to 4^th^ apical cell; 2^nd^ apical cell biggest. Hind wing with two cross veins.

Male sternal abdominal apodemes extending to distal margin of 4^th^ sternite.

**Male genitalia.** Pygofer side tall and divided into two or three small lobes caudally, upper lobe and central lobe with short rigid setae terminally, lower lobe deeply contracted, usually with long macrosetae. Subgenital plate elongate, triangular with a row of short fine setae subbasally to apex, with indistinct peg-like setae at apex. Paramere with basal part slim and central part broad, thereafter gradually tapered to apex, with a row of microsetae on outer margin. Connective trapezoidal. Aedeagus with short preatrium; dorsal apodeme well developed bifurcate apically; aedeagal shaft usually short with long and asymmetrical processes apically; gonopore apical.

#### Distribution.

Oriental and Palaearctic regions.

### ﻿Key to species (males) of the genus *Paracyba*

**Table:** 

1	Aedeagal shaft with two distal processes (Figs 60–62)	***P.biprocessa* sp. nov.**
–	Aedeagal shaft with three distal processes	**2**
2	Aedeagus with lateral process arising from mid-length of shaft; two other processes slightly arched, longest process bent to right	***P.akashiensis* (Takahashi)**
–	Aedeagus with lateral process arising near apex; other two processes relatively straight, longest process bent to left	**3**
3	Two apical processes of aedeagus twice as long as subapical process	***P.soosi* Dworakowska**
–	Two apical processes of aedeagus nearly equal in length to subapical process	***P.nopporensis* Matsumura**

### ﻿ Paracyba akashiensis

Taxon classificationAnimaliaHemipteraCicadellidae

(Takahashi)

3A28BB4C-2196-50E0-AE57-A5EB64865606

Zygina akashiensis Takahashi, 1928: 442; Dworakowska 1982: 148, figs 602–614.

#### Distribution.

China (Taiwan), Japan, Russia.

### ﻿ Paracyba soosi

Taxon classificationAnimaliaHemipteraCicadellidae

Dworakowska

DC8EFF3F-DBC8-5517-B496-3BB5125BABFB

Paracyba soosi Dworakowska, 1977: 41, figs 253–259.

#### Distribution.

China (Hunan), Vietnam.

### ﻿ Paracyba nopporensis

Taxon classificationAnimaliaHemipteraCicadellidae

(Matsumura)

910B9F1B-544B-5080-856D-35F48D846DBA

Typhlocyba nopporensis Matsumura, 1932: 100; Dworakowska 1982: 150, figs 615–621.

#### Distribution.

Japan, Russia.

### ﻿ Paracyba biprocessa sp. nov.

Taxon classificationAnimaliaHemipteraCicadellidae

356AEC4C-2AD4-5C81-B096-565C4ACAE9C3

http://zoobank.org/NomenclaturalActs/7504C225-8537-4115-9196-9783F7F4A8D4

Figs 23 , 27 , 31 , 35 , 55–62

#### Note.

Head with coronal suture extended to anterior margin (Fig. 23). Face yellow, eyes dark brown, thorax light brown (Figs 27, 35). Crown with anterior margin white; pronotum with lateral margin white, remainder covered with a continuous large ocher patch extending to distal end of clavus of forewing (Figs 23, 27); scutellum with apex orange (Fig. 27). Forewing light ocher with few light smoky patches in apical half, remaining part transparent except for white brochosome field.

Male sternal abdominal apodemes extending to middle of 5^th^ sternite (Fig. 55).

Pygofer side tall and bilobate caudally, upper lobe with short setae terminally, lower lobe with several fine moderately long setae (Fig. 56). Subgenital plate elongate, triangular with single macroseta near base, a row of short rigid setae adjacent row of fine setae laterally and two indistinct peg-like setae apically (Fig. 58). Paramere with row of microsetae on outer margin and row of sensorial pits on inner margin (Fig. 59). Connective trapezoidal, with central ridge (Fig. 57). Aedeagal shaft with two apical processes, in ventral view left process slim, S-shaped, right process short and straight (Figs 60–62).

**Figures 37–54.**
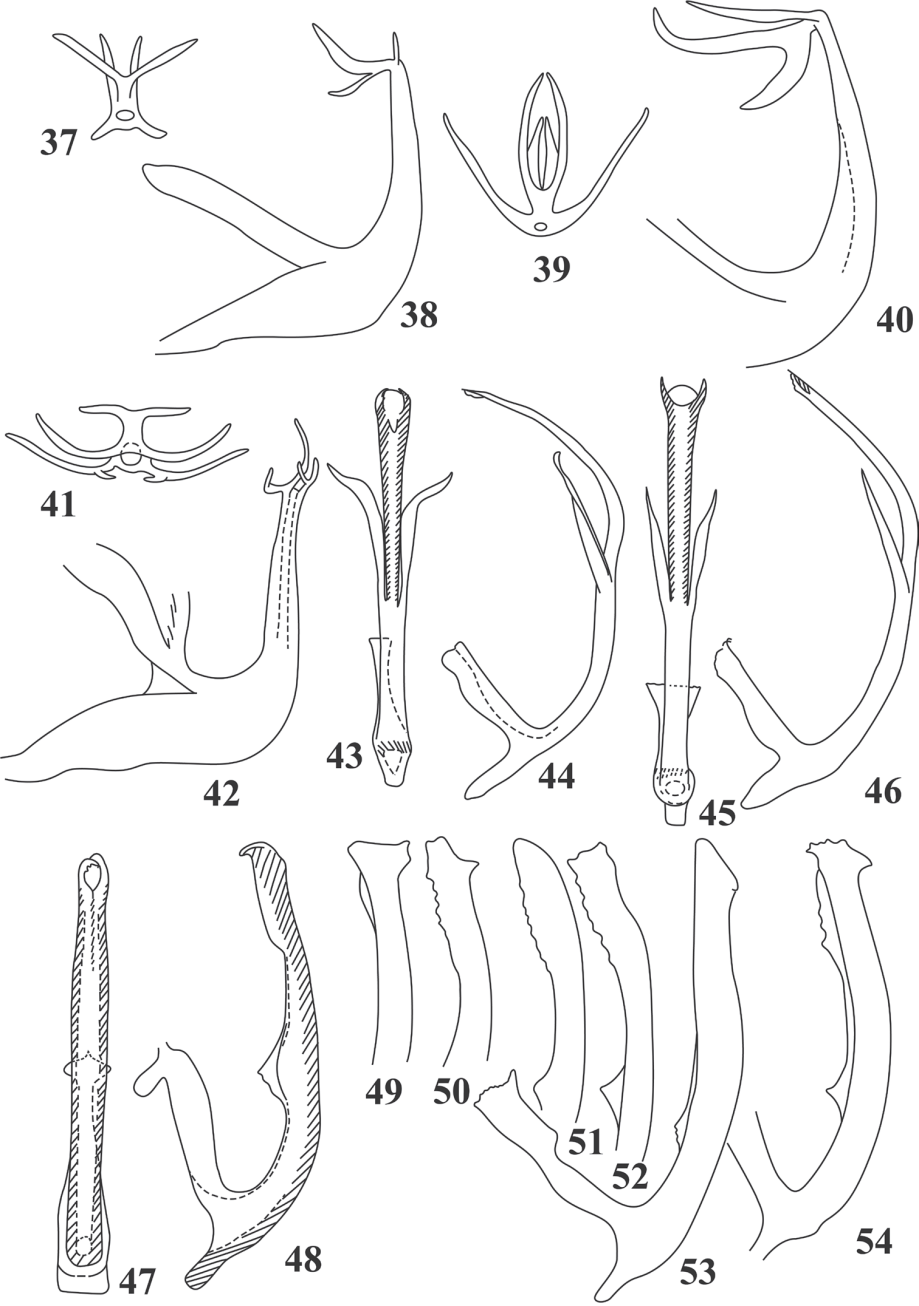
Male genitalia of Typhlocybini of China (after Dworakowska 1982) **37, 38***Edwardsianacorylicola***39, 40***E.praedestina***41, 42***E.singularis***43, 44***Hiratettixdistanti***45, 46***H.malaisei***47, 48***Linnavuorianaantiqua***49–54***L.malicola*.

#### Specimens examined.

***Holotype***: ♂, China, Shaanxi Province, Yangling, ix.1983, coll. Yalin Zhang. ***Paratypes***: 10♂♂, 4♀♀, same data as holotype.

#### Measurement.

Male, 3.1–3.3 mm (including wing).

#### Etymology.

This new species is named for the two aedeagal processes, rather than three in other species.

#### Remarks.

This new species resembles *Paracybasoosi* Dworakowska, 1977 in coloration and male genitalia, but it differs from the latter in aedeagal shaft having two rather than three distal processes and processes of very different length.

### ﻿ Shamala

Taxon classificationAnimaliaHemipteraCicadellidae

Dworakowska rec. nov.

F4AB70E5-9FC0-5B24-A4FD-BFE97FC0BC05

Shamala Dworakowska, 1980: 169, figs 174–187.

#### Type species.

*Shamalamikra* Dworakowska, 1980.

#### Remarks.

The genus *Shamala* was erected by Dworakowska (1980); thereafter she described four additional species from India and Nepal (1981), Sikkim (1994), and India (1982). In this paper, a new species, *S.annulata* sp. nov., from Yunnan, China, is described and illustrated which increases the number of valid species in this genus to six.

**Figures 55–62.**
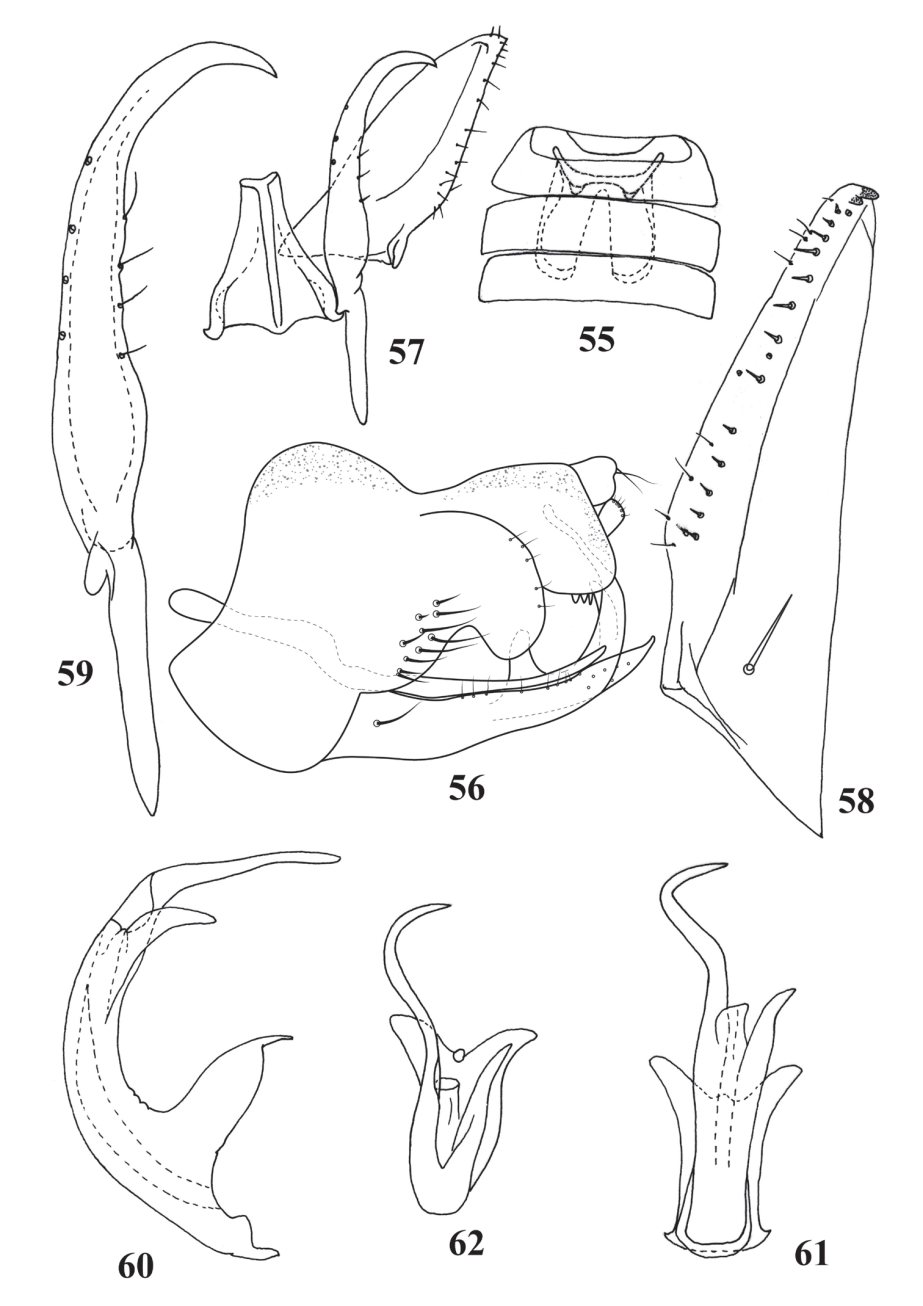
Male genitalia and sternal abdominal apodemes of *Paracybabiprocessa* sp. nov. **55** sternal abdominal apodemes **56** genitalia capsule, lateral view **57** paramere, connective and subgenital plate, dorsal view **58** subgenital plate **59** paramere **60** aedeagus, lateral view **61** aedeagus, posterior view **62** apical part of aedeagus, dorsal view.

#### Diagnosis.

Body slim and cream with occasional light brown patches. Head slightly wider than pronotum with length along midline slightly shorter than distance between eyes. Forewing parallel-sided, rounded terminally; RP and MP’ petiolate at base. Hind wing gradually narrowing from base to apex and rounded terminally with two cross veins.

Male sternal abdominal apodemes extending to 4^th^ or 5^th^ sternite.

**Male genitalia.** Genital capsule short; pygofer side with a weekly sclerotized area near middle of hind margin; several rigid setae at caudo-ventral angle and macrosetae on middle part. Subgenital plate gradually narrowing towards apex with a macroseta at base and row of short peg-like setae from middle to apex of outer margin; two rigid setae apically and few fine setae on inner margin subapically. Connective laminate, with stem well developed. Paramere with caudad part long, with several setae on outer margin. Aedeagal shaft with distal asymmetrical processes; with long membranous terminal part.

#### Distribution.

Oriental Region.

### ﻿ Shamala annulata sp. nov.

Taxon classificationAnimaliaHemipteraCicadellidae 

DF75CE9B-0334-59B5-A4D9-F4F7C2330266

http://zoobank.org/3853DE59-4325-4DFB-A39B-F3364B8B0CC4

Figs 24 , 28 , 32 , 36 , 63–71

#### Note.

Body cream (Fig. 24). Scutellum orange with basal triangles yellowish green (Fig. 28). Forewing bluish white with brownish patch on CuA’’.

Abdominal apodemes extending to distal margin of 4^th^ sternite.

**Figures 63–71.**
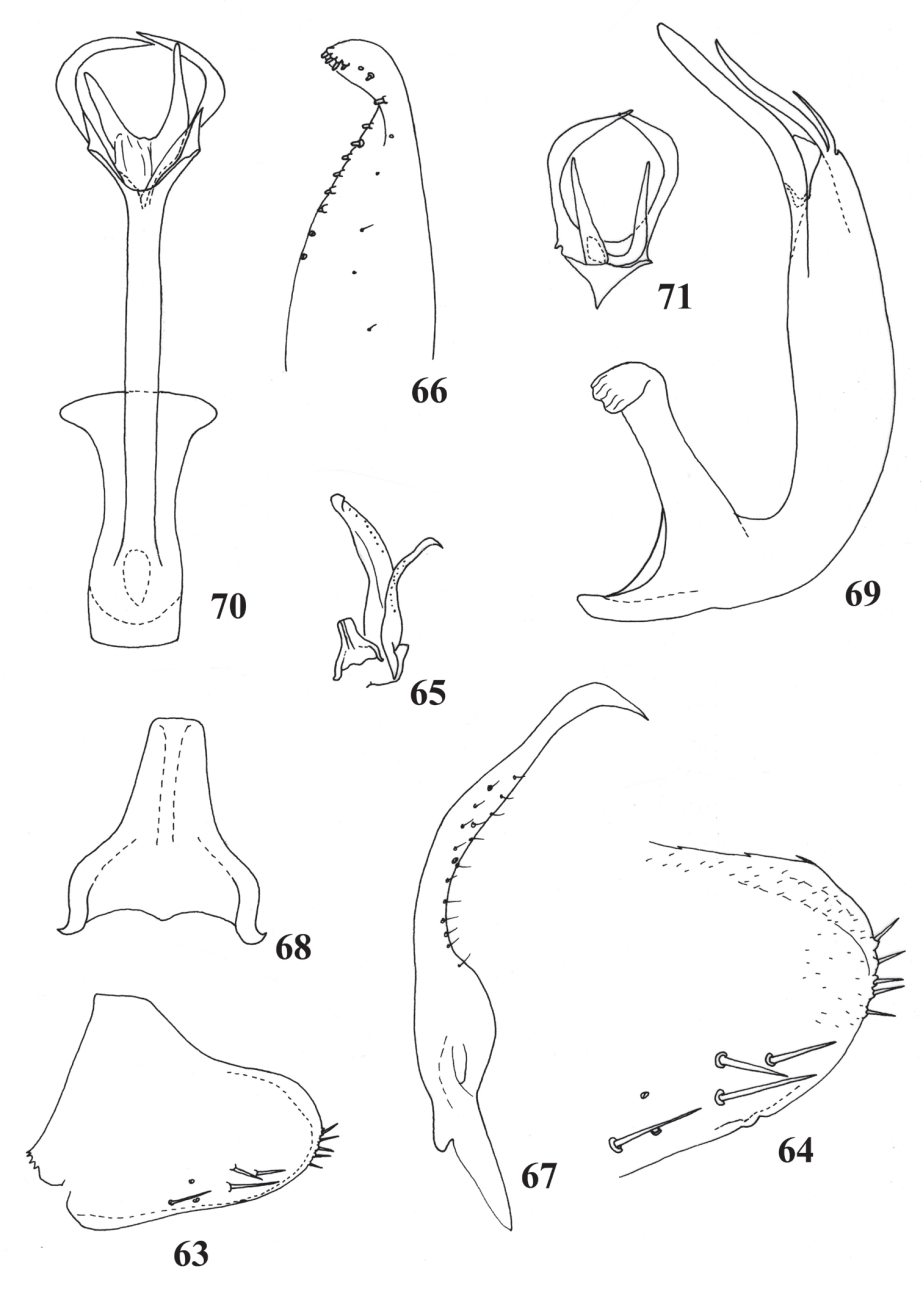
Male genitalia of *Shamalaannulata* sp. nov. **63, 64** pygofer side, lateral view **65** paramere, connective and subgenital plate, dorsal view **66** subgenital plate **67** paramere **68** connective **69** aedeagus, lateral view **70** aedeagus, posterior view **71** apical part of aedeagus, ventral view.

**Male genitalia.** Pygofer side with several macrosetae ventrally and rigid short setae terminally (Figs 63, 64). Subgenital plate long and narrow with row of short peg-like setae from middle part to apex on outer margin (Fig. 66). Connective slender with short central lobe, stem relatively long (Fig. 68). Aedeagal shaft with four asymmetrical processes apically, of which two dorsal processes are longer and curved inward forming a ring-like shape, two ventral processes straight and slightly divergent (Figs 69–71).

#### Specimens examined.

***Holotype***: ♂, China, Yunnan Province, Sanchahe, 7.vi.1991, coll. Rungang Tian. ***Paratypes***: 3♂♂, same data as holotype.

#### Measurement.

Male, 2.94 mm (including wing).

#### Etymology.

The name of this species is derived from the Latin word “annulus”, referring to the two dorsal processes of the aedeagal shaft forming a ring-like shape.

#### Remarks.

This new species resembles *Shamalaricasta* Dworakowska, 1981 in the structure of the male genitalia, but it differs from the latter by the two longer apical processes of aedeagal shaft forming a ring-like shape.

## Supplementary Material

XML Treatment for Edwardsiana

XML Treatment for Edwardsiana corylicola

XML Treatment for Edwardsiana ishidai

XML Treatment for Edwardsiana praedestina

XML Treatment for Edwardsiana rosae

XML Treatment for Edwardsiana singularis

XML Treatment for Hiratettix

XML Treatment for Hiratettix arisanellus

XML Treatment for Hiratettix distanti

XML Treatment for Hiratettix malaisei

XML Treatment for Hiratettix matsumurai

XML Treatment for Linnavuoriana

XML Treatment for Linnavuoriana antiqua

XML Treatment for Linnavuoriana malicola

XML Treatment for Paracyba

XML Treatment for Paracyba akashiensis

XML Treatment for Paracyba soosi

XML Treatment for Paracyba nopporensis

XML Treatment for Paracyba biprocessa

XML Treatment for Shamala

XML Treatment for Shamala annulata
